# Treating Children and Adolescents with Obesity: Predictors of Early Dropout in Pediatric Weight-Management Programs

**DOI:** 10.3390/children11020205

**Published:** 2024-02-05

**Authors:** Giovanni Luppino, Malgorzata Wasniewska, Celeste Casto, Chiara Ferraloro, Alessandra Li Pomi, Giorgia Pepe, Letteria Anna Morabito, Angela Alibrandi, Domenico Corica, Tommaso Aversa

**Affiliations:** 1 Department of Human Pathology of Adulthood and Childhood, University of Messina, Via Consolare Valeria 1, 98125 Messina, Italy; giovilup97@gmail.com (G.L.); celestecasto@libero.it (C.C.); chiarferri@gmail.com (C.F.); alessandra.lipomi92@gmail.com (A.L.P.); giorgia.pepe@unime.it (G.P.); domenico.corica@unime.it (D.C.); tommaso.aversa@unime.it (T.A.); 2 Pediatric Unit, AOU Policlinico G. Martino, Via Consolare Valeria 1, 98125 Messina, Italy; letteria.morabito@gmail.com; 3 Department of Economics, University of Messina, 98125 Messina, Italy; angela.alibrandi@unime.it

**Keywords:** adolescent, dropout, glucometabolic alterations, obesity, pediatrics

## Abstract

Background: Possible therapeutic failure of pediatric obesity is influenced by the high dropout rate. The aim of this study was to evaluate the rate of dropout and the rate of weight loss over the 24 months of follow-up. Methods: The retrospective, single-center study, involved 489 patients followed for obesity in the period 2016–2020. Patients’ auxological data and blood samples were collected during the first (V1) and last visit (V2). Dropout was defined as a follow-up of less than 12 months and/or including less than one visit every 6 months. Patients were divided into two groups and compared: Group A of dropout (297 patients) and Group B of non-dropout (192 patients). Results: In the follow-up period, which had a mean duration of 24 months, the dropout rate was 60.7%. In Group A, the percentage of patients with BMI ≥ 3 SD at V2 was significantly higher than that in Group B. In Group B, the percentage of patients with pathological HOMA-IR and with fasting glucose >100 mg/dL was higher than group A. The probability of dropout was positively associated with pubertal stage and negatively with impaired fasting glycemia and pathological insulinemia at V1. Conclusion: The study demonstrated a high dropout rate during follow-up, mainly among adolescents and patients with no glucometabolic alterations.

## 1. Introduction

The increased incidence of childhood obesity is one of the major public health issues worldwide [1]. The prevalence of obesity has doubled in more than 70 countries since 1980. In 2015, 107.7 million children were affected by obesity. According to the Organization for Economic Cooperation and Development (OECD), from 2020 to 2050, overweight and related diseases will reduce life expectancy by about three years in OECD, EU28, and G20 countries. The prevalence of obesity is lower in Italy than in other states, but it is at the highest level in children: one in three children in Italy is overweight [2].

Compared to the general population, children with obesity are at greater risk of developing chronic cardiovascular and metabolic diseases, such as arterial hypertension (AH), precocious atherosclerosis [1], dyslipidemia, impaired glucose tolerance, insulin resistance, type 2 diabetes mellitus (T2DM), and non-alcoholic fatty liver disease (NAFLD) [3]. The most serious complications of obesity are directly related to the age of obesity onset and to the percentage of weight excess [4,5]. Specifically, obesity leads to increased oxidative stress, which is linked to the pathogenesis of long-term cardiovascular and metabolic alterations [1]. Adiposity-related cardiovascular and glucometabolic complications are caused by several metabolic and inflammatory pathogenetic factors. Adipose cells produce metabolically active molecules implicated in molecular pathways of several inflammatory cascades, apoptosis, oxidative stress, and energy homeostasis [6,7]. Insulin resistance (IR) is an important link between obesity and associated cardiovascular and metabolic risk. [8]

Given the significant health and social consequences, both in the short- and long-terms, medical and scientific communities are implementing strategies for the prevention and treatment of childhood obesity. This has become even more important following the emergence of COVID-19 and the related safety procedures for containing the pandemic, which have had a negative impact on the lifestyle of children with overweight and obesity [9]. Knowledge of childhood obesity is extremely important to avoid the onset of long-term complications in adulthood or to promptly treat complications that may already occur in the pediatric age. Family histories of obesity and cardiometabolic disease are important risk factors for the early onset of obesity in childhood and are related to obesity severity [10]. Childhood obesity treatment is not limited to the reduction in caloric intake and the increase in energy expenditure, but it should aim at a permanent change in the living habits of the child and his or her family [11]. In clinical practice, a child nutrition plan should be individualized based on several elements: age, food preferences, cultural preferences, family and personal lifestyle, and concurrent medical diseases. Appropriate diet and physical activity programs should consider the age of patients and the severity of obesity complications, such as musculoskeletal system impairment. Several intervention strategies (behavioral therapy, cognitive behavioral therapy, and therapeutic education of the patient) require the active participation of parents and achieve better adherence to physical activity and diet [12,13]. However, this is made difficult by the high dropout rate during follow-up.

Pediatric weight-loss programs seem to yield moderate effectiveness and not all interventions demonstrate favorable outcomes. This could be attributed to elevated dropout rates and inadequate adherence within weight-loss programs, leading to a diminished impact of these interventions. Dropout leads to poor disease control and decreases the treatment’s effectiveness, eventually impacting health outcomes. Therefore, a prospective strategy to enhance the effectiveness of pediatric weight-management programs involves tackling dropout issues and promoting better compliance. Reducing dropout rates in lifestyle modifications in pediatric weight-control interventions is, therefore, critical for long-term behavioral changes [14,15,16].

The aim of this retrospective study was to evaluate (1) the dropout rate of patients with obesity during the follow-up, researching possible related factors, and (2) the percentage of weight loss at its end.

## 2. Materials and Methods

### 2.1. Study Design and Population

This was a retrospective, monocentric study conducted at the Pediatric Endocrinology department of the University Hospital of Messina, Italy. The study involved 489 children and adolescents who were followed in the clinic from January 2016 to January 2020 for overweight or obesity not attributed to genetic causes and in the absence of other diseases that could potentially contribute to weight gain.

As per international guidelines, overweight and obesity definitions rely on percentiles of the weight-to-length ratio or body mass index (BMI), considering variations in sex and age. From the age of 2, BMI is used, adhering to the WHO 2006 reference system [17], and is used up to the age of 5 with the WHO 2007 reference system [18]. Accordingly, children with overweight fall into a BMI SD range of 2 to 3, while those with obesity have a BMI SD of 3.

Inclusion criteria were as follows: (a) age at the first visit between 2 and 18 years, (b) diagnosis of obesity, (c) body mass index standard deviation (BMI SD) at the first visit ≥ 2 SD, (d) healthy full-term births, and (e) no other chronic medical conditions at obesity onset. Exclusion criteria were as follows: (a) diagnosis of endocrinology or genetic obesity, (b) preterm births, or (c) chronic medical condition or pharmacological therapies at enrolment in the study.

During the first visit, patients were offered a treatment plan based on a healthy and balanced Mediterranean diet and at least 20 min of moderate/intense physical activity per day [19,20]. To improve adherence, a family-based behavioral treatment was integrated [21]. Weight loss was assessed using the BMI standard deviation score, deeming a reduction in the BMI z score by 0.5 as the optimal outcome [22]. All patients and their families received specific multidisciplinary counseling (nutritionist, pediatric endocrinologist, psychologist, physical activity) at the first visit (V1) and at all follow-up visits for the 24 months of follow-up, to educate about proper diet and implement physical activity.

Since most treatment efficacy studies show short-term effects (6–12 months) [23], we defined “Dropout” as a follow-up of less than 12 months and/or fewer than 1 follow-up visit every 6 months throughout the follow-up. The follow-up period was primarily of 24 months with semestral visits. The dropout rate was evaluated during the first 12 months, to evaluate early dropout, and in the second half of follow-up. In addition, based on this criterion, to assess dropout rates and associated factors, patients were divided into two groups that were compared during the follow-up: Group A (dropout group), comprising 297 patients, and Group B (non-dropout group), comprising 192 patients.

To assess the percentage of weight loss, we computed the difference between the BMI SD value at the first visit (V1) and the BMI SD value at the last visit (V2). The decrease in weight was attributed to those patients who had achieved a positive change in BMI SD at the end of the follow-up.

### 2.2. Clinical Evaluation and Laboratory Assessment

Comprehensive family and personal histories were gathered to determine the presence or absence of familial obesity and potential complications, including arterial hypertension (AH), dyslipidemia, impaired glucose tolerance, insulin resistance, or type 2 diabetes mellitus (T2DM).

Auxological data were collected at V1 and V2. The following measurements were obtained with standard methods: height, weight, and BMI. Trained staff measured height and weight in children dressed in minimal underclothes without shoes. Body weight was measured with properly calibrated standard beam scales to the nearest 0.1 kg. Height was measured to the nearest 0.1 cm and standardized with a wall-mounted stadiometer. A thorough medical examination was then performed to assess pubertal development according to the Tanner classification [24]. At V1, patients with a pubertal stage of Tanner B/G 1 were considered prepubertal, those between B/G 2 and B/G 4 were considered pubertal, and complete pubertal development was considered in patients having the B/G5 stage. The assessment of the pubertal stage is vital for understanding dropout, especially in adolescence, where management strategies are more dependent on active patient participation and emerging patient autonomy may influence follow-up [25].

During V1, fasting blood samples were collected at least 8 h after the last meal for the determination of triglycerides, total and fractionated cholesterol, glucose, and insulin. These parameters were analyzed using standard techniques. Glucometabolic profile evaluation considered fasting values such as glucose > 100 mg/dL and HOMA-IR, calculated as ((fasting insulin (uU/mL) × fasting blood glucose (mg/dL)/405), which is pathological for values > 2.5 in prepubertal patients and > 4 in pubertal patients [26]. As defined by the National Cholesterol Education Panel, lipid status assessment considered values such as total cholesterol > 170 mg/dL, LDL > 130 mg/dL, HDL < 40 mg/dL, and triglycerides > 110 mg/dL [27] (Table 1).

### 2.3. Statistical Analysis

Data analysis was carried out retrospectively and completely anonymously. Numerical data are expressed as mean and SD, and categorical variables as numbers and percentages. Most of the examined variables were normally distributed as verified by the Kolmogorov–Smirnov test; consequently, comparison analysis between groups was conducted via Student’s *t*-test for numerical variables and via Pearson’s chi square test for categorical variables. To establish the possible association between weight loss or dropout (dependent variables) and the different analyzed variables (family history of obesity or its complications, sex, age and severity of obesity at the first visit, presence of metabolic alterations at the first visit, presence or absence of weight decrease, duration of follow-up), appropriate regression models were estimated. In particular, both univariate and multivariate stepwise logistic regression models were used to identify the factors significantly impacting the dichotomous outcomes. In addition, Cox regression analysis was employed for dropout to take into account the follow-up time. Statistical analyses were performed using SPSS 22.0 for Windows. A *p*-value of less than 0.05 was considered statistically significant.

## 3. Results

Out of 489 subjects, 260 (53.2%) were female. Family histories of obesity, DMT2, and arterial hypertension were positive in 41.3%, 39.1%, and 44.4% of subjects, respectively.

At V1, the mean age was 9.8 ± 2.9 years. BMI and BMI SD mean values were 29.7 ± 4.5 kg/m^2^ and 3.8 ± 1.2 SD, respectively. Three hundred and forty-nine patients (71.4%) had a BMI ≥ 3 SD and one hundred and forty (28.6%) had a BMI ≥ 2 SD and <3 SD. During this period, 91 subjects (18.6%) completed a follow-up of at least 12 months; 101 subjects (20.7%) completed a follow-up of at least 24 months.

**Figure 1:**
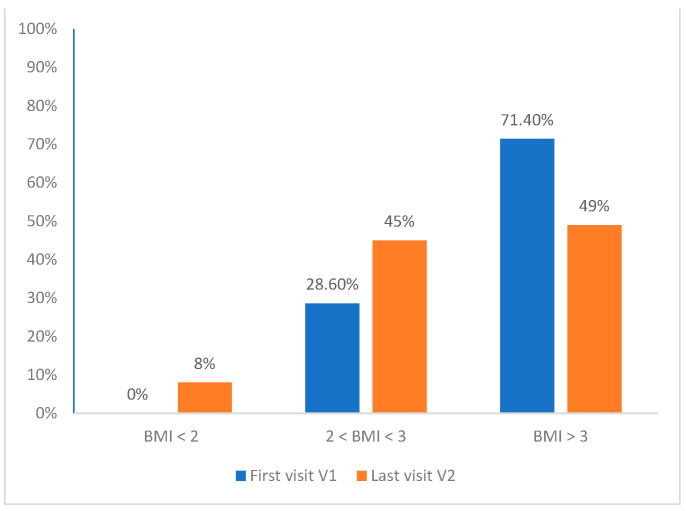
Variation in BMI DS between first (V1) and last visit (V2) in the entire group of patients: at first visit 71.4% of patients had a BMI ≥ 3 SD and 28.6% ≥ 2 SD and <3 SD; at the second visit we can see that many patients had achieved weight loss.

At V2, 7.4% had a BMI < 2 SD and 48.1% had a BMI ≥ 3 SD. BMI and BMI SD mean values were 29.88 ± 4.9 kg/m^2^ and 3.1 ± 1 SD, respectively. Data for the entire study population are summarized in Table 1 and Figure 1. BMI SDS values between V1 and V2 are significantly different (*p* < 0.0001) in the overall study population, but this includes both dropouts and those remaining in follow-up, making it unsuitable for assessing actual follow-up effectiveness.

### 3.1. Dropout Evaluation

To assess dropout rates and associated factors, the authors defined “Dropout” as a follow-up of less than 12 months and/or fewer than one follow-up visit every 6 months throughout the follow-up. Only 192 patients (Group B) completed follow-up, which had a mean duration of 20.8 ± 23.2 months (range 0.5–120 months). The lowest dropout rate was recorded between 12 and 23 months of follow-up: 91 patients (18.6%: Group B1) completed a follow-up of at least 12 months and 101 patients (20.7%: Group B2) completed a follow-up of at least 24 months. The dropout rate at 12 months was 47.4% (232 patients) and increased to 60.7% (297 patients: Group A) by the end of the observation period. No statistically significant differences were found in anamnestic variables, gender, and age between Group A and Group B. Additionally, no statistically significant differences were found in pubertal stage and BMI SD at the first visit. Analytical data of the two groups (A and B) are summarized in Table 2 and Table 3.

Both groups were uniform for age, gender, and BMI SDS at the first visit. However, BMI SD and the percentage of patients with a BMI ≥3 SD at V2 in Group A were significantly higher (*p* = 0.002; *p* = 0.027, respectively) than those in Group B. Additionally, the percentage of patients in advanced pubertal stage (age range 10.7–17.24 years) in Group A was higher than that in Group B, although not statistically significantly (15.2% vs. 8.9%, *p* > 0.05). Furthermore, the percentage of patients with pathological HOMA-IR was significantly higher in Group B (51.1% vs. 41.3%; *p* = 0.036), as was the percentage of subjects with fasting glucose values > 100 mg/dL (16.5% vs. 8.9%; *p* = 0.012). Within group B, patients who followed a longer follow-up (Group B2) had a lower age and a higher BMI SDS at V1, compared to those patients who left the follow-up before 24 months (Group B1).

Stepwise multivariate regression analysis showed a positive association between dropout probability and pubertal stage at V1 (OR 1.39) and a negative association between dropout probability and fasting glucose > 100 mg/dL at V1 (OR 0.52) (Table 4). These results were confirmed by a Cox regression model that provided evidence of a positive association between dropout probability and pubertal stage at V1 (HR 1.43) and a negative association between dropout probability and fasting insulin of 15 uUI/mL > 100 mg/dL at V1 (HR 0.98) (Table 5). These findings suggest that the probability of dropout is higher in adolescents and in the absence of obesity glucometabolic complications (Table 4).

### 3.2. Weight Loss Evaluation

In Figure 1, it is evident that at V2, 307 patients achieved weight loss. Specifically, out of the 140 patients with a BMI ≥ 2 SD and <3 SD at V1, only 30 experienced a reduction in BMI. Among patients with a BMI < 3 SD at V1, this percentage dropped to 1.7% (Table 6).

The BMI modification displayed in Figure 1 between the first and last visit highlights that a considerable proportion of patients with severe obesity (BMI ≥ 3 SD) achieved weight loss. Patients with severe obesity who successfully reduced their BMI had a more extended and consistent follow-up. In fact, at the last visit, the percentage of patients with BMI ≥ 3 SD was significantly higher in the dropout group than in the non-dropout group (*p* = 0.027) (Table 3).

Logistic regression analysis revealed a negative association between weight loss and age > 10 years and a positive association between weight loss and nonpathological HOMA-IR (Table 7). Conversely, variables such as a positive family history of obesity or its complications, BMI at V1, alterations in lipid status, or fasting glucose did not exert a significant influence on weight variation.

## 4. Discussion

Obesity is one of the most significant problems in society and the change in lifestyle linked to the COVID-19 epidemic seems to have worsened the situation [28].

According to the Global Burden of Disease Study, risks associated with high body mass index (BMI) increased by more than 25% between 1990 and 2015. The prevalence of obesity has doubled in more than 70 countries since 1980. A total of 107.7 million children had obesity in 2015. According to the Organization for Economic Co-operation and Development (OECD), from 2020 to 2050 overweight and related diseases will reduce life expectancy by about three years in OECD, EU28, and G20 countries. OECD data show that obesity is less common in Italy than in other states in the OECD area, but it is at the highest levels in children. In fact, one in three children in Italy are overweight. The data collected showed that overweight children perform less well in school and show lower life satisfaction [2,8].

It is commonly known that childhood obesity causes complications in adulthood such as diabetes and various cardiovascular problems, with high costs for public health and a reduction in quality of life [29]. Additionally, a family history of obesity, DM2, arterial hypertension, and dyslipidemia is associated with earlier and more severe forms of obesity [8]. Moreover, risk of developing T2DM and cardiovascular disease is more elevated in children with obesity, complicated by a significant alteration in insulin sensitivity than peers without IR, given the same BMI [30].

Unfortunately, data from the literature regarding the long-term outcomes of intervention programs according to lifestyle modifications (balanced diet and implementation of physical activity) in children and adolescents with obesity report highly variable results. Dropout rates are generally quite high, negatively affecting the success in the treatment of these patients. In the different studies, the percentage of patients who dropped out of follow-up at 12 months varies between 7% and 43%; this percentage rises to 52% when evaluated after 24 months [31].

The present study results revealed a high level of dropout in the population examined. This means that the treatment with the proposed change in the lifestyle of children and adolescents living with obesity fails to lead to a satisfactory result due to poor adherence. Above all, there is probably a persistent lack of perception of obesity in developmental age as the cause of chronic disease in adulthood by patients and their caregivers. The results of this study indicate that social education programs on this issue need to be significantly increased in local communities [32].

Moreover, data regarding the efficacy of obesity treatments have revealed that children with parents with obesity and/or siblings show less weight reduction during follow-up, which could be partly explained by genetic factors [33]. However, an important role must be ascribed to psychosocial factors, which appear to be independent predictors of the success or failure of therapeutic behavioral interventions. Parental involvement has been recognized as one of the most important factors in successful behavioral treatments during both childhood and adolescence [34,35]. It has been amply demonstrated that multifactorial interventions may be more effective if they involve the family, are delivered in specialist settings, and combine changes in lifestyle habits, particularly diet and physical activity (generally involving behavioral management techniques) [36].

Consistent with data from the literature, the present study demonstrated a high dropout rate (60.7%). The analysis pointed out some interesting new findings: adolescents are at higher risk of disregarding controls, and this correlates with greater difficulty in losing weight. This finding is probably related to the chronicity of obesity and obesogenic behaviors such as pronounced tendency to digital addiction and junk food intake. Instead, the presence of glucometabolic alterations at the beginning of the follow-up makes dropping out less likely; this could be due to increased family awareness of the risks of not achieving weight loss. In the present study, no association was found between the severity of obesity at the first visit and the dropout rate.

These findings could confirm the need for adequate information for patients and their caregivers about the presence of the first possible complications of childhood obesity, calling it “unhealthy obesity” to decrease dropout and increase “therapeutic success” [37]. Factors such as family history of obesity or its complications, gender, or severity of obesity at first visit did not influence dropout and long-term outcomes in the patient group examined. It is also well known that the positive family history of obesity and its metabolic and cardiovascular complications could greatly affect the early onset and severity of overweight [9,38].

Elevated dropout rates and inadequate adherence lead to a diminished impact of clinical interventions into weight loss and lifestyle change. Dropout leads to poor disease control and decreases the treatment’s effectiveness, eventually impacting health outcomes. Therefore, a prospective strategy to enhance the effectiveness of pediatric weight-management programs involves tackling dropout issues and promoting better compliance. Reducing dropout rates in lifestyle modifications in pediatric weight-control interventions is, therefore, critical for long-term behavioral changes. In the literature, different studies have aimed to identify variables associated with compliance and dropout of a multidisciplinary weight-loss intervention program.

In a prospective longitudinal cohort study of children with overweight and obesity in socially deprived areas of Rotterdam, Hassan et al. [14] demonstrated a dropout rate of 33.9%. Children with overweight parents had a significantly lower probability of dropout than those with parents with obesity or normal weight. It is possible to speculate that normal-weight parents worry less about weight, so they may not evaluate the urgency of losing weight. Instead, the higher risk of dropout found in the group of children with parents with obesity could be correlated with the notion that necessary lifestyle changes might be more complicated for them [14,39].

Park et al. [15] identified lower family functioning, lower initial attendance rates, and non-self-directed pathways as factors associated with dropout. Lower initial attendance rates and a poor-quality family relationship during early follow-up have also been linked to higher rates of early and late dropout. Developing a supportive family environment and addressing factors early in the intervention may help reduce overall dropout rates in obesity-prevention programs. Emphasis on the family environment, including family function, is crucial and underscores the need to tailor the program from the beginning to reduce dropout rates. Knowledge of the adolescent’s circumstances and family environment must be the priority for a successful intervention with sustained adherence [15].

Other different psychosocial and demographic predictors could be implicated in poor compliance and high dropout rates. Jensen et al. showed that lower gross family income, distance between the participant’s home and the treatment site, and self-revelation of depressive symptoms by the youth were associated with a lower rate of attendance at the follow-up visit [16].

However, family background variables and characteristics could predict success in a family-based lifestyle-intervention program. Pott et al. [33] found that three characteristics of the child’s family predicted success in the weight-loss intervention program: maternal depression, maternal avoidant attachment attitude, and obese siblings. Mental disease of mothers might not be able to help the child sufficiently in her/his efforts to observe treatment and change eating and physical activity habits. Maternal avoidant attachment style is associated with a less satisfying patient–provider relationship and poor metabolic control. The presence of a brother with obesity who is not participating in the program and continues to adhere to unhealthy eating and sedentary habits may demotivate the primary child in their attempts to modify those behaviors [33].

Prado et al. [40] compared adherence and dropout rates among adolescents with obesity engaged in a behavioral counseling intervention, with or without recreational physical activity. The study found a lower dropout rate among adolescents who received physical activity treatment. Consequently, incorporating a recreational physical activity component into a non-intensive behavioral intervention appears to be a viable strategy for reducing dropout rates in adolescents with obesity and in treatment. In addition, it is recommended that each family member actively participate in the physical activity program to enhance lifestyle changes and achieve positive outcomes [40].

Barlow et al. [41] reported that the degree of obesity at the start of treatment was the only important factor for retention rate, while Zeller [42] et al. found that age at the start of treatment was the dependent factor for not dropping out. These results are in line with the high risk of dropout in adolescents without glucometabolic changes at the start of follow-up highlighted by the present study. Danielsson et al. [43] found a significant dropout rate, especially in the adolescent group, with only 30% of subjects remaining after 3 years. As also highlighted in the present study, this evidence suggests a massive dropout rate in the early phase of follow-up.

A prospective strategy to enhance the effectiveness of pediatric weight-management programs involves tackling dropout issues and promoting better compliance. Improved adherence to follow-up of patients with obesity might be possible by raising awareness among parents, who should be more involved in all parts of the program from the first visit. Reducing the dropout rate of lifestyle modifications in pediatric weight-control interventions is therefore critical to achieving long-term behavioral changes. Future research should be focused on ways to identify patients at risk of dropping out of treatment and to develop new strategies to reduce the dropout rate.

Moreover, the possibility of personalized therapy, recently discussed in the literature, could be the best way to improve the management of obese young people, decreasing dropout and improving longitudinal outcomes [44]. However, due to the persistent high dropout rate in adolescents with obesity yet still without serious complications, it is important to improve prevention methods and to ensure the chronicity and severity of obesity are fully known. In summary, understanding and analyzing the factors influencing the duration of follow-up and dropout in children or adolescents living with obesity is critical to reducing the likelihood of failure of obesity prevention and treatment strategies.

## 5. Conclusions

The present study highlighted a high dropout rate in the population examined. In this study, adolescents and patients with advanced obesity at the onset were more at risk of abandonment. Moreover, the percentage of subjects with familiarity of obesity was also higher in the dropout group. This seemed to suggest, in part, attributing considerable responsibility to the lack of awareness of the family, which does not consider obesity as a pathology unless there were already glycol-metabolic complications at the onset of follow-up.

In accordance with the above, patients with impaired HOMA-IR at the onset of follow-up showed a significantly higher rate of weight loss than the general population. This trend was not seen in patients older than 10 years, who probably showed even less interest in the pathology.

In conclusion, adolescents and subjects without any glucometabolic alterations at the onset of follow-up need closer monitoring and possible counseling to avoid early abandonment and the further development of complications in adulthood. Placing greater attention on the problem of childhood obesity and making families responsible for correct prevention and disease management would also allow for the progression of medical research in this area.

## Figures and Tables

**Table 1:** Auxological and biochemical data of the entire study population at the first visit, V1. (FH: family history).

Variable	Percent
M	46.8%
F	53.2%
2 ≤ BMI < 3 DS	28.6%
BMI ≥ 3 DS	71.4%
Prepubertal	57.7%
Pubertal	29.7%
Complete pubertal development	12.7%
FH of obesity	41.3%
FH of T2DM	44.4%
Pathological HOMA-IR	45.1%
Total cholesterol > 170 mg/dL	41.8%
Triglycerides > 110 mg/dL	17.4%
Fasting glucose > 100 mg/dL	11.9%
Fasting insulin > 15 uUI/mL	41.4%

**Table 2:** Data comparison between Group A (dropout) and Group B (non-dropout).

Variable	Group A (Mean ± SD)	Group B (Mean ± SD)	*p* Value
Age V1 (years)	9.90 ± 2.83	9.73 ± 3.08	>0.05
BMI V1 (kg/m2)	29.93 ± 4.51	29.47± 4.56	>0.05
BMI SD V1	3.798 ± 1.27	3.78 ± 1.23	>0.05
Follow-up duration (months)	16.10 ± 25.20	28.26 ± 17.53	0.002
Age V2 (years)	11.37 ± 2.79	12.16 ± 2.93	0.003
BMI V2 (kg/m2)	30.13 ± 5.04	29.49 ± 4.75	>0.05
BMI SD V2	3.28 ± 1.01	2.97 ± 1.07	0.002

**Table 3:** Categorical variable comparison between Group A (dropout) and Group B (non-dropout).

Variable	Group A (n 297)	Group B (n 192)	*p* Value
M	44.8%	50%	n.s.
F	55.2%	50%	n.s.
2 ≤ BMI < 3 SD (V1)	27.3%	30.7%	n.s.
BMI ≥ 3 SD (V1)	72.7%	69.3%	n.s.
BMI < 2 SD (V2)	4.0%	12.5%	n.s.
2 ≤ BMI < 3 SD (V2)	42.4%	47.4%	n.s.
BMI ≥ 3 SD (V2)	53.2%	40.1%	0.027
Prepuberal	56.2%	59.9%	n.s.
Puberal	28.6%	31.2%	n.s.
Complete pubertal development	15.2%	8.9%	n.s.
FH of obesity	43.8%	37.5%	n.s.
FH of T2DM	38.7%	39.6%	n.s.
Pathological HOMA-IR	41.3%	51.1%	0.036
Total cholesterol > 170 mg/dL	43.0%	39.9%	n.s.
Triglycerides > 110 mg/dL	17,4%	17.4%	n.s.
Fasting glucose > 100 mg/dL	8.9%	16.5%	0.012
Fasting Insulin > 15 uUI/mL	38.2%	46.3%	n.s.

**Table 4:** Stepwise multivariate regression analysis for pubertal stage, pathological fasting glucose.

Variables	OR	I.C. 95%	*p* Value
Advanced pubertal stage	1.39	1.04–1.85	0.022
Fasting glucose > 100 mg/dL	0.52	0.29–0.93	0.028
Fasting insulin > 15 uUI/mL	0.64	0.93–0.96	0.032

**Table 5:** Cox regression analysis for pubertal stage, pathological fasting glucose.

Variables	HR	I.C. 95%	*p* Value
Advanced pubertal stage	1.43	1.21–1.69	0.001
Fasting glucose > 100 mg/dL	0.99	0.98–1.01	0.281
Fasting insulin > 15 uUI/mL	0.98	0.96–0.99	0.003

**Table 6:** Variation in BMI DS between first (V1) and last (V2) visit in the entire group of patients, divided on the basis of severity of obesity.

	BMI < 2 DS at V2	2 ≤ BMI < 3 at V2	BMI ≥ 3 DS at V2
2 ≤ BMI < 3 DS at V1	21.4%	70.7%	7.9%
BMI ≥ 3 DS at V1	1.7%	34.1%	64.2%

**Table 7:** Logistic regression analysis for age and HOMA-IR classes.

Variables	OR	I.C.95%	*p*
Age > 10 anni	0.57	0.39–0.83	0.003
Nonpathological HOMA-IR	1.50	1.05–2.21	0.025

## Data Availability

Data are available from the authors upon request. The data are not publicly available due to privacy reasons.
